# Exploratory Modeling of Postoperative Atrial Fibrillation After Cardiac Surgery with Cardiopulmonary Bypass Using Inflammatory Biomarkers and Clinical-Surgical Factors

**DOI:** 10.3390/medsci14050513

**Published:** 2026-08-25

**Authors:** Rosa Michel Martínez-Contreras, Marina María de Jesús Romero-Prado, Karla Mayela Bravo-Villagra, Aneth Karine Sánchez-Soto, Eliseo Portilla-de Buen, Guillermo Alejandro Muñoz-Benavides, Ramón Arreola-Torres, José Marco Medina-Carrillo, Jorge Straffon-Castañeda, Joel Regalado-Silva, Ana Rebeca Jaloma-Cruz

**Affiliations:** 1 Doctorado en Genética Humana, Centro Universitario de Ciencias de la Salud, Universidad de Guadalajara, Guadalajara 44340, Jalisco, Mexico; michelmartinezc@gmail.com (R.M.M.-C.); karla.bravo2318@alumnos.udg.mx (K.M.B.-V.); 2 División de Genética, Centro de Investigación Biomédica de Occidente, Instituto Mexicano del Seguro Social, Guadalajara 44340, Jalisco, Mexico; 3 Instituto de Biología Molecular en Medicina y Terapia Génica, Departamento de Fisiología, Centro Universitario de Ciencias de la Salud, Universidad de Guadalajara, Guadalajara 44340, Jalisco, Mexico; marina.rprado@academicos.udg.mx; 4 Instituto de Nutrigenética y Nutrigenómica, Centro Universitario de Ciencias de la Salud, Universidad de Guadalajara, Guadalajara 44340, Jalisco, Mexico; 5 Facultad de Medicina, Universidad Autónoma de Guadalajara, Zapopan 45129, Jalisco, Mexico; 6 División de Investigación Quirúrgica, Centro de Investigación Biomédica de Occidente, Instituto Mexicano del Seguro Social, Guadalajara 44340, Jalisco, Mexico; 7 Hospital General de Topilejo, Secretaría de Salud de la Ciudad de México, Mexico City 14500, Mexico; 8 Servicio de Cardiocirugía, Unidad Médica de Alta Especialidad del Hospital de Especialidades, Centro Médico Nacional de Occidente, Instituto Mexicano del Seguro Social, Guadalajara 44340, Jalisco, Mexico

**Keywords:** atrial fibrillation, biomarkers, cardiac surgery procedures, cardiopulmonary bypass, inflammation mediators, risk factor

## Abstract

Background/Objectives: Postoperative atrial fibrillation (POAF) is a common complication after cardiac surgery with cardiopulmonary bypass (CPB), increasing morbidity and prolonging hospitalization. This study aimed to develop and validate an exploratory prediction model that integrates perioperative inflammatory biomarkers with clinical and surgical variables to identify patients at risk of early POAF. Methods: A prospective exploratory cohort of 89 patients undergoing coronary artery bypass grafting (CABG; *n* = 36), valve surgery (*n* = 40), or CABG–valve surgery (*n* = 13) was evaluated. Clinical, surgical, and proinflammatory serum biomarkers (IL-6, IL-8, IL-10, and CRP) were recorded preoperatively (T1) and at 24 h (T2) and 48 h (T3) postoperatively. Multiple-comparison adjustments were made using the Benjamini–Hochberg false discovery rate. Predictor selection was based on bootstrap-derived stability using LASSO-penalized logistic regression, and the final model was estimated using Firth’s bias-reduced logistic regression. Results: POAF incidence was 8.3% in CABG, in contrast to 22.5% and 30.8% in valve and CABG-valve surgeries, respectively. After multiple-comparison corrections, only IL-6 at T2 postoperatively was significantly higher in patients who subsequently developed POAF. Bootstrap-based stability selection retained T2 postoperative IL-10 and magnesium concentrations in the final model, which achieved an apparent AUC of 0.776 and a bootstrap optimism-corrected AUC of 0.728, with acceptable calibration (Brier score = 0.103), negligible multicollinearity (VIF = 1.04), and a negative predictive value of 95.5% at the optimal Youden threshold. Conclusions: Our findings support an exploratory prediction model with moderate discrimination for POAF after cardiac surgery with CPB, providing a methodological foundation for future multicenter validation studies.

## 1. Introduction

Cardiovascular diseases (CVDs) remain the leading cause of morbidity and mortality worldwide. These conditions impose a substantial public health burden because of their significant clinical and socioeconomic consequences [1,2]. Conditions such as coronary artery disease, valvular heart disease, heart failure, and arrhythmias often require surgical intervention in advanced stages [1]. Cardiac surgery has markedly improved patient survival and quality of life. However, it is frequently associated with postoperative complications, notably postoperative atrial fibrillation (POAF), the most common sustained arrhythmia. POAF occurs in 20–50% of patients, depending on the procedure and patients’ clinical status. It is linked to longer hospitalizations, thromboembolic events, heart failure, and increased mortality, all of which contribute to higher healthcare costs [3,4,5,6].

POAF results from a combination of preoperative vulnerability, surgical stress, and postoperative disturbances [7]. Key risk factors include advanced age, male sex, hypertension, diabetes, heart failure, structural atrial abnormalities, and higher surgical risk scores. Intraoperative factors, such as prolonged cardiopulmonary bypass time and atrial manipulation, also increase risk [1,7]. Current clinical and surgical risk scores have limited predictive value for POAF, underscoring the need for better tools [4].

Evidence increasingly supports the central role of inflammation in POAF pathophysiology, especially after cardiac surgery with cardiopulmonary bypass (CPB) [5,6,8]. Surgical trauma, ischemia–reperfusion injury, contact with extracorporeal circuits, and endothelial activation trigger systemic inflammation. This inflammation is characterized by cytokines, acute-phase proteins, and oxidative stress mediators [7,8,9]. These changes promote atrial electrical and structural remodeling, alter ion-channel function, impair conduction, and create a transient proarrhythmic substrate. This substrate favors atrial fibrillation, particularly in the first 48–72 h post-surgery [5,8,10].

Cytokines such as interleukin-6 (IL-6), interleukin-8 (IL-8), interleukin-10 (IL-10), and C-reactive protein (CRP) have been studied as biomarkers of POAF [11,12,13,14]. IL-6 and IL-8 are proinflammatory cytokines that mediate neutrophil recruitment, endothelial activation, and myocardial dysfunction. IL-10 reflects anti-inflammatory mechanisms within the overall response [12,13,15]. CRP, an acute-phase reactant, is linked to cardiovascular risk and to atrial fibrillation in both surgical and non-surgical settings [16,17]. However, prior studies report inconsistent findings. Most focus on single biomarkers, single-time measurements, or small groups, without integrating clinical and surgical factors [13,18,19].

The timing of inflammatory biomarker changes can reveal mechanisms underlying POAF. Early postoperative cytokine surges, followed by either rapid resolution or persistent elevation, may reflect varying degrees of inflammation, compensatory responses, and atrial vulnerability [20]. However, integrated multimodal approaches that combine dynamic inflammatory biomarkers, perioperative metabolic variables, and clinical-surgical factors, along with internal validation strategies, remain limited, particularly in Latin American populations undergoing cardiac surgery with cardiopulmonary bypass [4,18].

This study aimed to assess the predictive value of inflammatory biomarkers (IL-6, IL-8, IL-10, and CRP) measured at multiple time points, in combination with clinical and surgical factors, for predicting POAF in patients undergoing cardiac surgery with cardiopulmonary bypass. By integrating dynamic inflammatory biomarkers with clinical, surgical, and postoperative metabolic variables, we sought to develop an exploratory multimodal model for early POAF risk stratification and to evaluate the feasibility of this integrative approach in a Latin American cohort [21].

## 2. Materials and Methods

### 2.1. Study Design and Population

We conducted a prospective, analytical, longitudinal cohort study from June 2022 to December 2025. Consecutive patients aged 50 years or older who underwent cardiac surgery with cardiopulmonary bypass (CPB) and provided informed consent were enrolled. Recruitment took place at the Cardiac Surgery Department of the High Specialty Medical Unit (UMAE), Hospital de Especialidades, National Medical Center of the West (IMSS) in Guadalajara, Jalisco, Mexico. Eligible procedures included coronary artery bypass grafting (CABG), valve replacement (aortic, mitral, and/or tricuspid), and combined CABG with valve replacement. Patients were referred from the state of Jalisco and other regions of western Mexico.

### 2.2. Ethical Issues

The study was conducted in accordance with the Declaration of Helsinki and was approved by the Institutional Review Board “National Committee of Scientific Research” (CNIC, IMSS), composed of the Ethics, Biosecurity, and Research committees, with approval report registered as F-CNIC-2022-019, dated 29 June 2022.

### 2.3. Inclusion and Exclusion Criteria

Patients without a history of atrial fibrillation (AF), as confirmed by clinical records, were included. The absence of AF was further verified by a 12-lead electrocardiogram and a 12- to 24-h preoperative Holter monitor (Holter Model TLC5000, Contec, Qinhuangdao, Hebei, China).

Exclusion criteria included hemoglobinopathies, acute or chronic renal or hepatic disease, autoimmune diseases, coagulation disorders, heparin-induced thrombocytopenia, asthma, active malignancies, or inability to obtain preoperative baseline samples.

**Figure 1:**
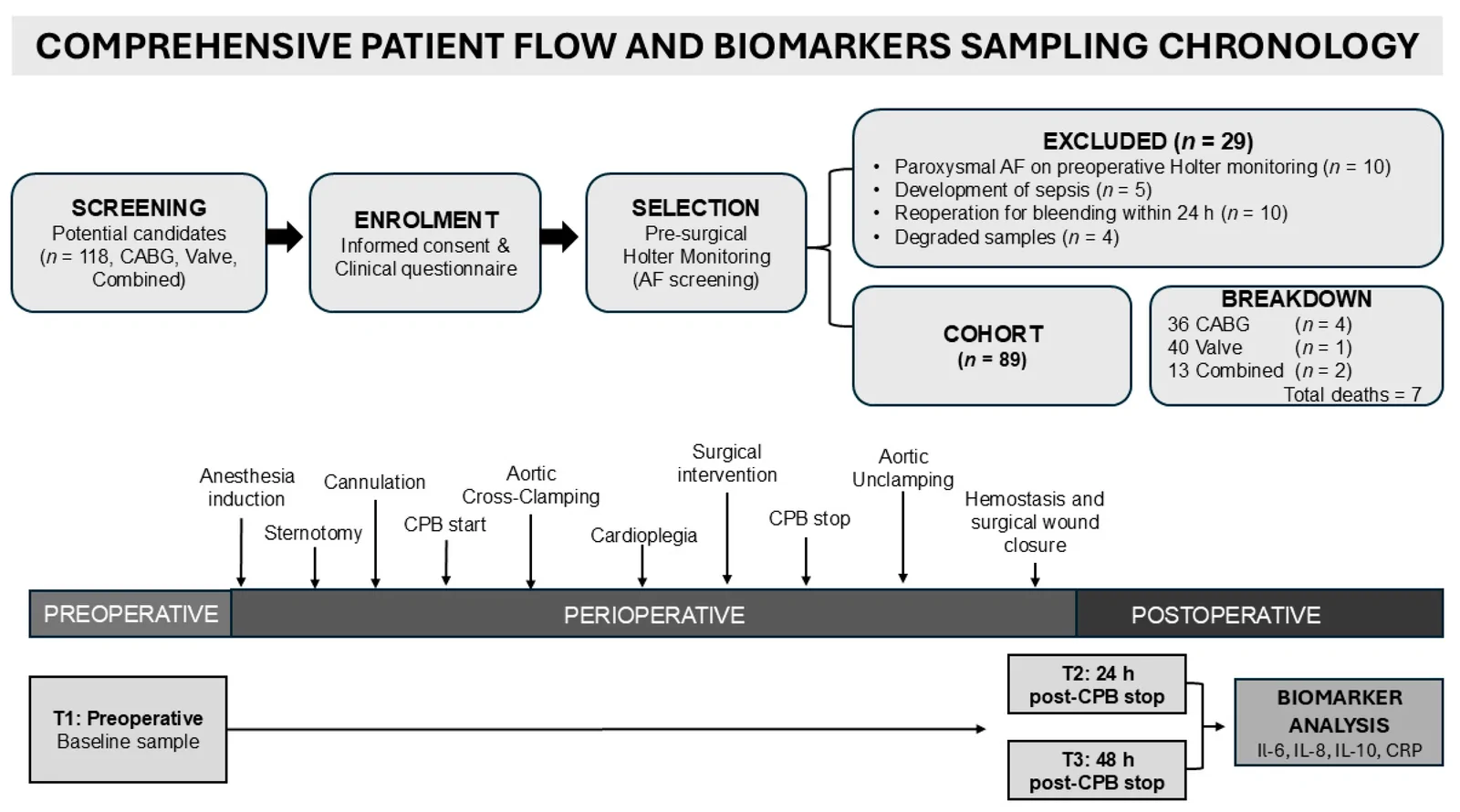
Methodology flowchart. The upper diagram outlines the patient enrollment process, types of cardiac surgery, and selection criteria for assembling the patient cohort. The bottom diagram details the sampling timeline throughout the surgical process and the inflammatory biomarker analysis. CABG: Coronary Artery Bypass Grafting; Valve: Valve replacement surgery; Combined: CABG-Valve surgery; AF: Atrial Fibrillation; T1: Patient plasma sampling at preoperatory; T2: Patient plasma sampling at 24 h postoperative; T3: Patient plasma sampling at 48 h postoperative; IL-6: Interleukin-6; IL-8: Interleukin-8; IL-10: Interleukin-10; CRP: C-Reactive Protein.

During follow-up, patients were excluded if collected samples were inadequate or insufficient for analysis, if they withdrew informed consent, or if they developed major intraoperative or postoperative complications. Major complications included severe CPB-related events, surgical reintervention for bleeding, requirement for an intra-aortic balloon pump, or infectious or inflammatory complications (Figure 1).

Death during follow-up was not an exclusion criterion; all data collected up to the time of death were included in the analysis.

### 2.4. Clinical, Laboratory, and Surgical Data Collection

Clinical data were collected using a standardized questionnaire that included demographic characteristics, cardiovascular diagnoses, comorbidities, anthropometric measurements, and left ventricular ejection fraction (LVEF).

Laboratory parameters were measured preoperatively and at 24 h postoperatively, including hemoglobin, hematocrit, platelet count, leukocyte count, glucose, urea, creatinine, electrolytes (calcium, sodium, potassium, chloride, and magnesium), and total protein.

Surgical variables included CPB duration, aortic cross-clamp time, number of coronary grafts or valves replaced, type of prosthesis used, initial and final activated clotting time (ACT), and perioperative blood loss volume.

### 2.5. Anesthesia and Surgical Procedures

All patients underwent preoperative anesthetic evaluation and perioperative monitoring, including electrocardiography, noninvasive blood pressure, peripheral oxygen saturation, and anesthetic depth assessment. After induction of general anesthesia and orotracheal intubation, invasive arterial line monitoring was established for arterial blood gas analysis at multiple perioperative stages and for measurement of plethysmographic and morphometric arterial pressure patterns. Central venous access was used for administration of vasoactive and cardiovascular drugs, central venous pressure (CVP) measurement, and central venous blood gas analysis as needed. Anesthesia was maintained with inhaled agents and then transitioned to total intravenous anesthesia during cardiopulmonary bypass (CPB).

Post-CBG management, including hemodynamic support, reversal of anticoagulation, and hemostasis control measures, was performed according to each patient’s clinical needs and in accordance with institutional protocols. All medications and blood products administered during the perioperative period were systematically recorded and analyzed as independent variables (see Supplementary Material, Table S7).

Surgeries were performed by different surgeons. All procedures were performed via median sternotomy using a standardized sequence: CPB initiation, aortic cross-clamping, cardioplegia administration, surgical intervention, aortic unclamping, CPB weaning, hemostasis, drain placement, and wound closure. Procedures included aortic, mitral, and tricuspid valve replacements (mechanical or biological prostheses as appropriate) and coronary artery bypass grafting (CABG) using internal mammary arteries, radial artery, and/or saphenous vein grafts. In combined procedures, CABG was performed before valve surgery.

### 2.6. Blood Sampling and Biomarker Analysis

Peripheral blood samples were collected at three predefined time points: before surgery as baseline (T1), at 24 h after surgery (T2), and 48 h after surgery (T3) (Figure 1). This temporal nomenclature (T1–T2–T3) is used consistently throughout the analysis and results.

No samples were collected during surgery to avoid analytical bias from CPB-induced hemolysis. The analysis focused on samples collected between 24 and 48 h postoperatively (T2 and T3), which corresponds to the peak of the acute inflammatory response. Serum concentrations of interleukins IL-6, IL-8, and IL-10 were measured using a multiplex immunoassay (Bio-Plex Pro™ Human Cytokine 8-Plex Panel, catalog M50000007A, Bio-Rad Laboratories, Hercules, CA, USA) based on Luminex xMAP^®^ magnetic bead technology. Samples were processed according to the manufacturer’s protocol and quantified using five-parameter calibration curves. The assay’s limits of detection (LOD) were 2.6 pg/mL for IL-6, 1.0 pg/mL for IL-8, and 0.3 pg/mL for IL-10. According to the manufacturer, intra-assay coefficients of variation were 7%, 9%, and 5%, respectively, and inter-assay coefficients of variation were 11%, 4%, and 6%, respectively.

Serum C-reactive protein (CRP) levels were measured by a latex particle-enhanced immunoturbidimetric assay (BioSystems, Cat. No. 31321, Barcelona, Spain) on a Mindray^®^ BS-200 automated analyzer (Mindray Bio-Medical Electronics Co., Shenzhen, China). According to the manufacturer, the method’s detection limit was 1.9 mg/L, with an analytical range of 1.9 to 150 mg/L. Intra-assay and inter-assay coefficients of variation were 2.9% and 2.6%, respectively.

To minimize variability and control for batch effects, all serial samples from each patient (T1, T2, and T3) were analyzed on the same plate or within the same analytical run. Although samples were not analyzed in duplicate due to sample and budget constraints, assay accuracy was ensured by meeting expected values for calibration curves, standards, and internal controls in each run.

Samples with non-quantifiable results were reviewed and found not to meet quality criteria for analysis, so they were excluded. As a result, serum samples from four patients were excluded from the final analysis, as detailed in the methodological flowchart (Figure 1).

### 2.7. Outcome Definition and Follow-Up

To confirm baseline sinus rhythm, all patients underwent a comprehensive preoperative evaluation that included a 12-lead electrocardiogram (ECG) and Holter monitoring. Postoperative atrial fibrillation (POAF) was defined as any episode of atrial fibrillation lasting at least 30 s.

During the postoperative period, all patients received continuous cardiac telemetry monitoring in the intensive care unit (ICU). After transfer to the general ward, cardiac monitoring continued per standard clinical protocols. POAF detection was guided by clinical presentation and confirmed by daily reviews of medical and nursing records from all shifts. Suspected arrhythmic events were verified by the attending physician using a 12-lead ECG.

Importantly, all blood samples were frozen and analyzed simultaneously at the end of the study, ensuring that clinical staff responsible for diagnosing POAF remained strictly blinded to biomarker results. Patient follow-up ended at hospital discharge.

### 2.8. Statistical Analysis

Descriptive statistics summarized clinical characteristics. Continuous variables were reported as mean ± standard deviation or as median (interquartile range), as appropriate, and categorical variables as frequencies and percentages. Normality was assessed with the Shapiro–Wilk test. Group comparisons used Student’s *t*-test or Mann–Whitney U test for continuous variables and χ^2^ or Fisher’s exact test for categorical variables. Because inflammatory biomarker distributions were markedly right-skewed, values were log2-transformed before regression modeling, allowing interpretation of odds ratios per doubling of concentration. Comparisons across biomarkers and time points were corrected for multiplicity using the Benjamini–Hochberg false discovery rate (FDR) procedure [22]. Because postoperative biomarkers could temporally coincide with or follow arrhythmia onset, comparisons at each postoperative time point were restricted to patients who remained free of POAF up to that time point (landmark approach) [23].

Associations between inflammatory biomarkers, clinical and surgical variables, and POAF were evaluated using Spearman’s correlation and univariate binary logistic regression. Given the limited number of POAF events (*n* = 16) relative to the number of candidate predictors (*n* = 50), backward stepwise selection was avoided because of its well-documented instability and optimism in small samples [24,25]. Instead, variable selection for the multivariable model was performed via stability selection: LASSO-penalized logistic regression, tuned by cross-validation, was refit across 300 bootstrap resamples, and predictors were retained based on selection frequency and effect-direction consistency across resamples, in combination with clinical relevance and biological plausibility [26]. Multicollinearity among candidate predictors was negligible (maximum VIF = 1.04). Missing data were minimal across candidate predictors; the only variable with missing observations was hypothyroidism status, which was unrecorded in 5.62% of patients. Analyses were performed using complete cases for the variables included in each model.

The final model included two predictors, yielding an events-per-variable ratio of 8.0. Given the study’s exploratory nature and the limited number of outcome events, the model was estimated using Firth’s penalized likelihood approach to reduce small-sample bias [27]. A three-predictor sensitivity model was also evaluated but not adopted as the primary model, given minimal performance improvement at the cost of a lower events-per-variable ratio. The final model was estimated using Firth’s bias-reduced (penalized maximum likelihood) logistic regression, which is appropriate for the limited number of events in this cohort. Consequently, coefficient estimates and effect sizes should be interpreted with caution and treated as exploratory until confirmed in larger, independent cohorts.

Receiver operating characteristic (ROC) curves with 95% confidence intervals (DeLong method) were used to assess the discriminative ability of individual and combined variables. Internal validation was performed using a full bootstrap procedure (1000 replicates), with variable selection repeated within each resample, yielding an optimism-corrected bootstrap estimate of model discrimination rather than validating only the fixed final model. Model calibration was assessed using calibration plots, apparent calibration slope and intercept, the Brier score, and the Hosmer–Lemeshow test [28,29]. Clinical utility was assessed using decision curve analysis [30]. The incremental predictive value of inflammatory biomarkers beyond clinical variables was evaluated using DeLong’s test for correlated ROC curves, the likelihood ratio test, and net reclassification improvement (NRI) and integrated discrimination improvement (IDI).

Statistical analyses were conducted in R (version 4.x) using the packages logistf, glmnet, pROC, rms, and nricens for model fitting, variable selection, discrimination, calibration, and reclassification analyses, respectively. A two-sided *p*-value < 0.05 was considered statistically significant, except where corrected for multiplicity as noted above.

## 3. Results

### 3.1. Baseline Clinical, Surgical, and Biochemical Characteristics According to POAF Status

Among the 89 patients included in the study, 16 developed postoperative atrial fibrillation (POAF), for an overall incidence of 17.9%. By surgical procedure, POAF occurred in 3 of 36 patients undergoing isolated coronary artery bypass grafting (CABG; 8.3%), 9 of 40 undergoing isolated valvular surgery (22.5%), and 4 of 13 undergoing combined CABG–valvular surgery (30.8%) (Supplementary Table S1). Regarding the timing of POAF onset, one patient developed the arrhythmia within approximately 24 h after surgery, nine between 24 and 48 h, and six after 72 h.

Baseline demographic and clinical characteristics by POAF status are summarized in Table 1. Exploratory comparisons of inflammatory biomarker concentrations between isolated CABG and isolated valve surgery are presented separately in Supplementary Table S2 and were not included in the primary comparison between patients with and without POAF.

Among the evaluated baseline characteristics, hypothyroidism was significantly more prevalent among patients who developed POAF than among those who did not (31.3% vs. 5.5%, *p* = 0.0018). No significant differences were observed for previous myocardial infarction, type 2 diabetes mellitus, systemic arterial hypertension, obesity, dyslipidemia, age, sex, or body mass index.

Surgical, hematologic, and biochemical variables are summarized in Table 2. No significant between-group differences were observed for cardiopulmonary bypass duration, aortic cross-clamp duration, activated clotting time, perioperative blood loss, EuroSCORE II, or left ventricular ejection fraction. Among postoperative laboratory parameters, only hemoglobin measured at 24 h differed significantly, with lower concentrations in patients who subsequently developed POAF than in those without POAF (9.80 vs. 10.8 g/dL, *p* = 0.007). No other hematologic or biochemical variables showed statistically significant between-group differences.

### 3.2. Inflammatory Biomarkers: Cytokines and C-Reactive Protei

Perioperative concentrations of IL-6, IL-8, IL-10, and C-reactive protein (CRP) by POAF status were analyzed. Because POAF could occur before postoperative blood collection, a landmark analysis was performed to preserve the temporal relationship between biomarker measurement and arrhythmia onset. Accordingly, only patients who remained free of POAF before each scheduled blood collection were included in the comparison for that postoperative time point.

Based on the exact timing of POAF onset, 12 of the 16 patients who subsequently developed POAF remained eligible for the 24-h landmark analysis, whereas only nine remained eligible for the 48-h analysis.

Because predictor selection was based on bootstrap stability rather than univariable significance testing, biomarkers that did not remain significant after FDR correction were still included in multivariable model development. However, after Benjamini–Hochberg false discovery rate correction, only IL-6 at 24 h remained statistically significant (median 12.44 vs. 4.97 pg/mL; unadjusted *p* = 0.012; FDR-adjusted *p* = 0.046), whereas the remaining biomarker comparisons no longer reached statistical significance.

**Figure 2:**
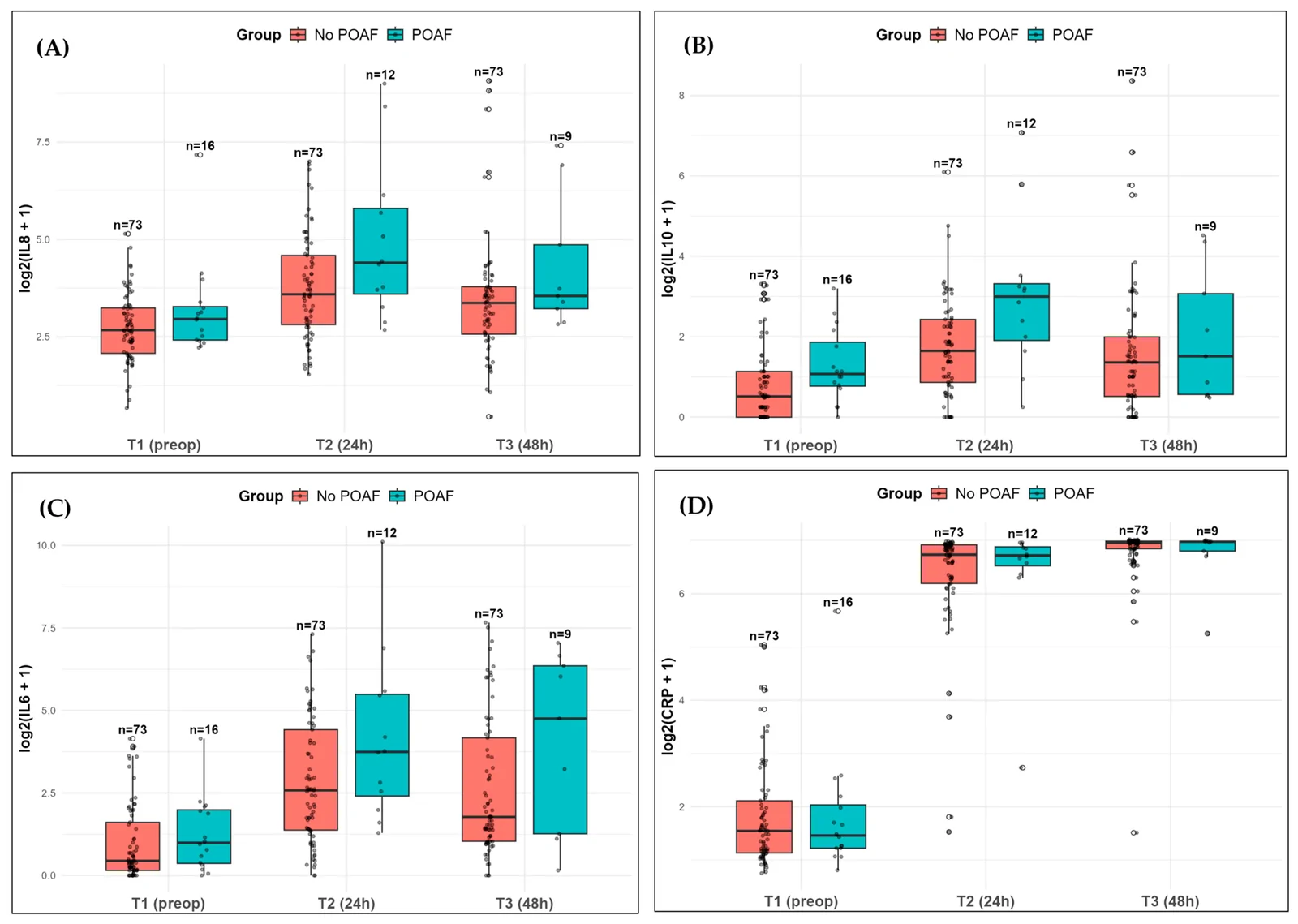
Perioperative distributions of inflammatory biomarkers by postoperative atrial fibrillation status. Box plots show log_2_-transformed concentrations of (**A**) IL-8, (**B**) IL-10, (**C**) IL-6, and (**D**) CRP measured preoperatively (T1) and at 24 h (T2) and 48 h (T3) after surgery. Boxes represent the interquartile range, horizontal lines indicate the median, whiskers extend to 1.5 times the interquartile range, and points represent individual observations. Postoperative plots include only patients who remained free of POAF before blood collection at each landmark time. CRP = C-reactive protein; IL = interleukin; POAF = postoperative atrial fibrillation.

The temporal distribution of IL-6, IL-8, IL-10, and CRP concentrations is shown in Figure 2. Median cytokine concentrations increased substantially at 24 h after surgery in both groups, although IL-6, IL-8, and IL-10 reached higher levels among patients who subsequently developed POAF. CRP concentrations also increased after surgery in both groups, reflecting the expected postoperative inflammatory response. Statistical comparisons of these distributions are summarized in Table S3.

As an exploratory secondary analysis, inflammatory biomarker concentrations were also compared by surgical procedure. Patients undergoing combined CABG–valvular surgery were excluded from this exploratory analysis because of the limited sample size and greater heterogeneity of this subgroup. Therefore, this analysis should be interpreted descriptively. As shown in Supplementary Table S2, IL-8 (T1) and IL-10 (T1) concentrations were significantly higher in patients undergoing valvular surgery than in those undergoing CABG (*p* = 0.010 and *p* = 0.027, respectively), whereas IL-6 showed no significant difference between groups (*p* = 0.068). No significant differences in IL-6 (T1), IL-8 (T1), or IL-10 (T1) concentrations were observed between surgical procedures at T2 or T3 after surgery. CRP concentrations were comparable between groups, except for a borderline difference at 24 h (*p* = 0.050).

Overall, the greatest differences between patients who developed POAF and those who remained free of the arrhythmia were observed during the early postoperative inflammatory response, particularly at 24 h after surgery. Therefore, these biomarkers were prioritized as candidate predictors for the subsequent multivariable modeling.

Because variable selection for predictive modeling was based on bootstrap stability selection rather than on univariable statistical significance alone, biomarkers that did not remain significant after FDR correction were not excluded a priori from subsequent multivariable analyses.

### 3.3. Variable Selection and Predictive Model Development

A total of 50 candidate variables spanning the demographic, clinical, surgical, hematologic, biochemical, and inflammatory biomarker domains were initially considered (Supplementary Table S4).

Because only 16 POAF events were observed, conventional multivariable variable-selection procedures, such as backward stepwise regression, were considered likely to yield unstable estimates and were therefore not applied. Instead, predictor stability was evaluated using LASSO-penalized logistic regression with cross-validation across 300 bootstrap resamples. For each candidate predictor, both the selection frequency and the consistency of the estimated direction of association were recorded.

Hypothyroidism had one of the highest bootstrap selection frequencies. However, because only nine patients had this condition, the corresponding regression estimates were deemed insufficiently stable for inclusion in the primary predictive model. Therefore, hypothyroidism was retained as an exploratory finding rather than as a component of the final model.

EuroSCORE II was also excluded because the estimated direction of its association with POAF varied across bootstrap resamples, indicating insufficient stability despite its clinical relevance. Consequently, EuroSCORE II was retained only as an exploratory finding.

The Society of Thoracic Surgeons (STS) score was not included in the final model because the available STS estimates were procedure-specific and were not developed to estimate the risk of postoperative atrial fibrillation. Consequently, combining procedure-specific STS scores in a single prediction model could introduce additional heterogeneity and complicate the interpretation.

Although IL-10 at 24 h was no longer statistically significant after false discovery rate correction, it showed high bootstrap selection stability and a consistently positive association, supporting its inclusion in the multivariable predictive model.

Log_2_-transformed IL-10 and magnesium concentrations at 24 h were ultimately retained as predictors in the primary model. Their bootstrap selection frequencies were 66.4% and 49.7%, respectively, and the direction of association remained consistent in 89.9% and 75.7% of bootstrap resamples. Complete bootstrap selection frequencies for all candidate predictors are presented in Supplementary Table S5.

### 3.4. Final Predictive Model

Univariable analyses of the candidate predictors are presented in Supplementary Table S4. These analyses were exploratory and served only as descriptive input for the subsequent bootstrap-based stability-selection procedure, rather than as the basis for predictor selection. The final two-predictor model was fitted using Firth’s bias-reduced logistic regression to reduce small-sample estimation bias. With 16 POAF events and two predictors, the events-per-variable ratio yielded an EPV of 8. Multicollinearity was negligible, with a variance inflation factor (VIF) of 1.04 in the final model.

The estimated linear predictor for the probability of POAF was:

logit[P(POAF)] = −5.630 + 0.585 × log_2_(IL-10 at 24 h + 1) + 1.149 × magnesium at 24 h

Both variables selected by the bootstrap stability procedure remained positively associated with the estimated probability of POAF in the final Firth logistic regression model (Table 3). A doubling of IL-10 concentration at 24 h was associated with the estimated odds of POAF (OR = 1.79, 95% CI: 1.20–2.90; *p* = 0.0046). Magnesium concentration at 24 h showed a positive association with POAF (OR = 3.15, 95% CI: 0.90–11.88), but this association did not reach conventional statistical significance (*p* = 0.0717). The predictive performance and internal validation of this model are presented in the following section.

### 3.5. Model Performance

**Figure 3:**
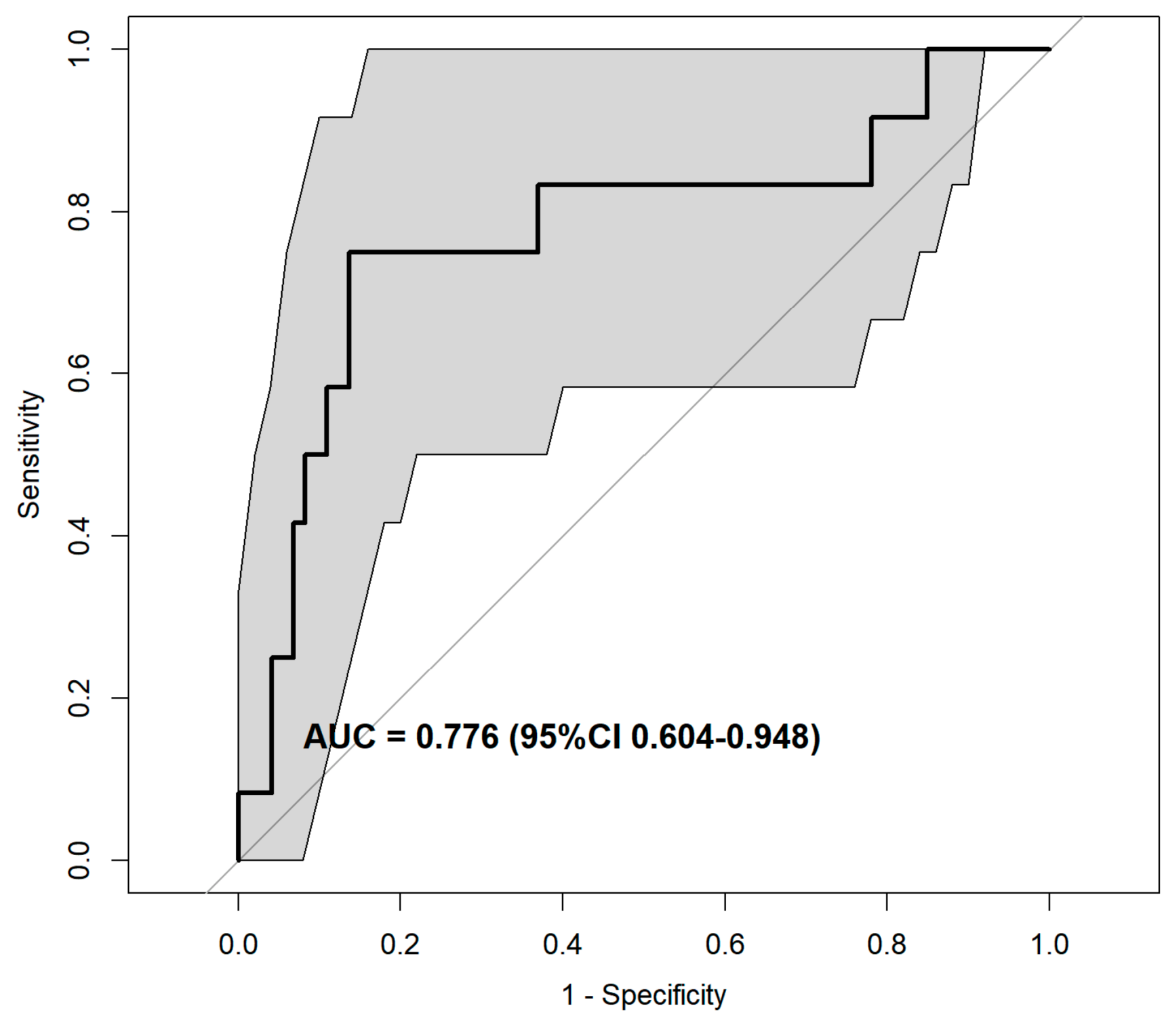
Receiver operating characteristic curve for the final postoperative atrial fibrillation model. The model included log_2_-transformed IL-10 and magnesium concentrations at 24 h and was fitted using Firth’s bias-reduced logistic regression. The solid line represents the receiver operating characteristic curve, and the shaded area represents the 95% confidence band. The apparent area under the curve was 0.776 (95% CI: 0.604-0.948). AUC = area under the curve; CI = confidence interval; IL-10, interleukin-10.

The discrimination performance of the final model is summarized in Figure 3. The apparent area under the receiver operating characteristic (ROC) curve (AUC) was 0.776 (95% CI: 0.604–0.948). Internal validation was performed using 1000 bootstrap replicates, with the complete predictor-selection procedure repeated within each resample to account for model-selection uncertainty. The mean bootstrap apparent AUC was 0.836, and the estimated optimism was 0.108, yielding an optimism-corrected AUC of 0.728.

Using the Youden-optimal predicted-probability threshold (0.191), the model achieved a sensitivity of 75.0%, specificity of 86.3%, positive predictive value of 47.4%, and negative predictive value of 95.5% (Table 4).

**Figure 4:**
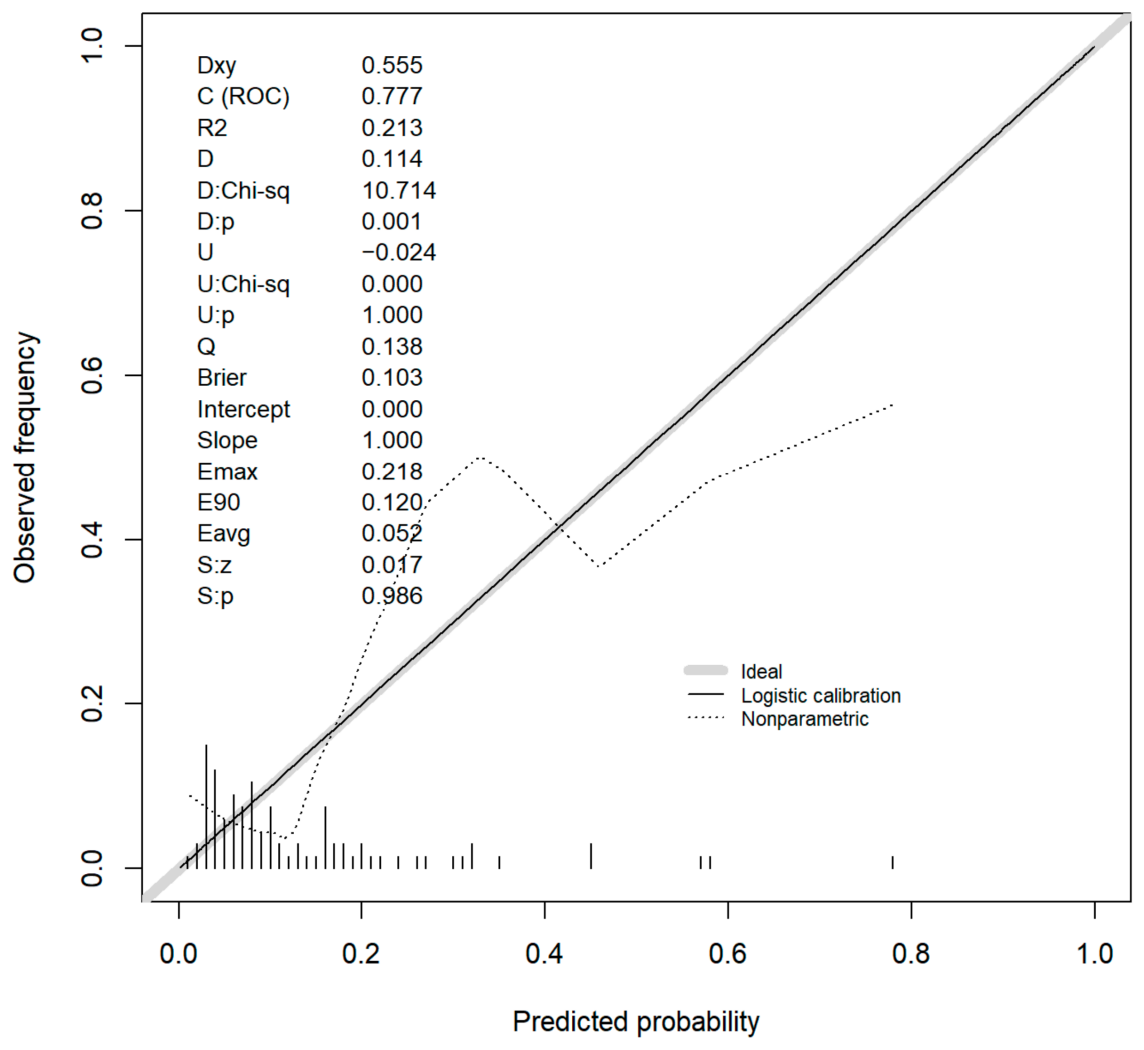
Calibration plot for the final postoperative atrial fibrillation model. Predicted probabilities are plotted against observed POAF frequencies. The diagonal line indicates perfect calibration; the solid line is the fitted logistic calibration curve, and the dotted line is the nonparametric calibration estimate. Rug marks show the distribution of predicted probabilities. Calibration metrics indicate good agreement between predicted and observed probabilities. POAF = postoperative atrial fibrillation.

Model calibration was satisfactory. The apparent calibration slope was 1.00, the calibration intercept was 0.00, and the Brier score was 0.103 (Figure 4). The Hosmer–Lemeshow goodness-of-fit test showed no evidence of poor calibration (χ^2^ = 1.00, df = 3, *p* = 0.801).

**Figure 5:**
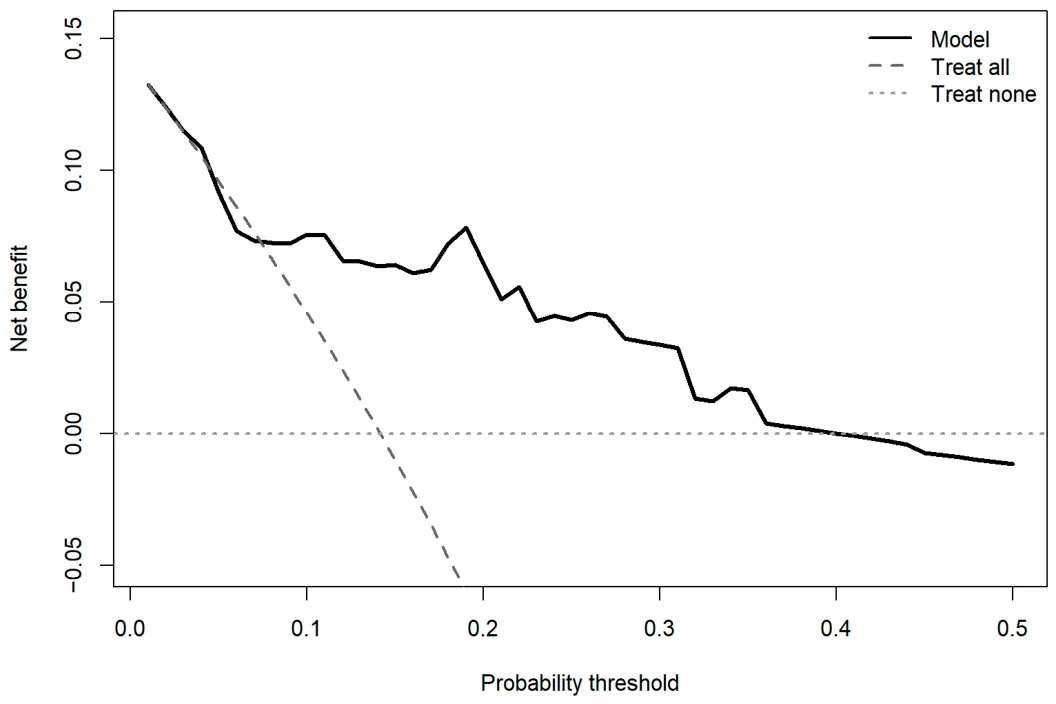
Decision curve analysis of the final postoperative atrial fibrillation model. Net benefit is shown across a range of predicted-probability thresholds. The solid line represents the final model, the dashed line the treat-all strategy, and the horizontal dotted line the treat-none strategy. The model showed greater net benefit than the reference strategies at thresholds of approximately 0.10 to 0.40.

Decision curve analysis showed that the final model had a greater net benefit than the treat-all and treat-none strategies across predicted-probability thresholds ranging from approximately 0.10 to 0.40 (Figure 5).

The decrease from the apparent to the bootstrap optimism-corrected AUC indicates moderate overfitting, which is expected in a prediction model developed from a relatively small dataset. These findings should be considered exploratory because external validation has not yet been conducted.

### 3.6. Comparison of Alternative Prediction Models

**Figure 6:**
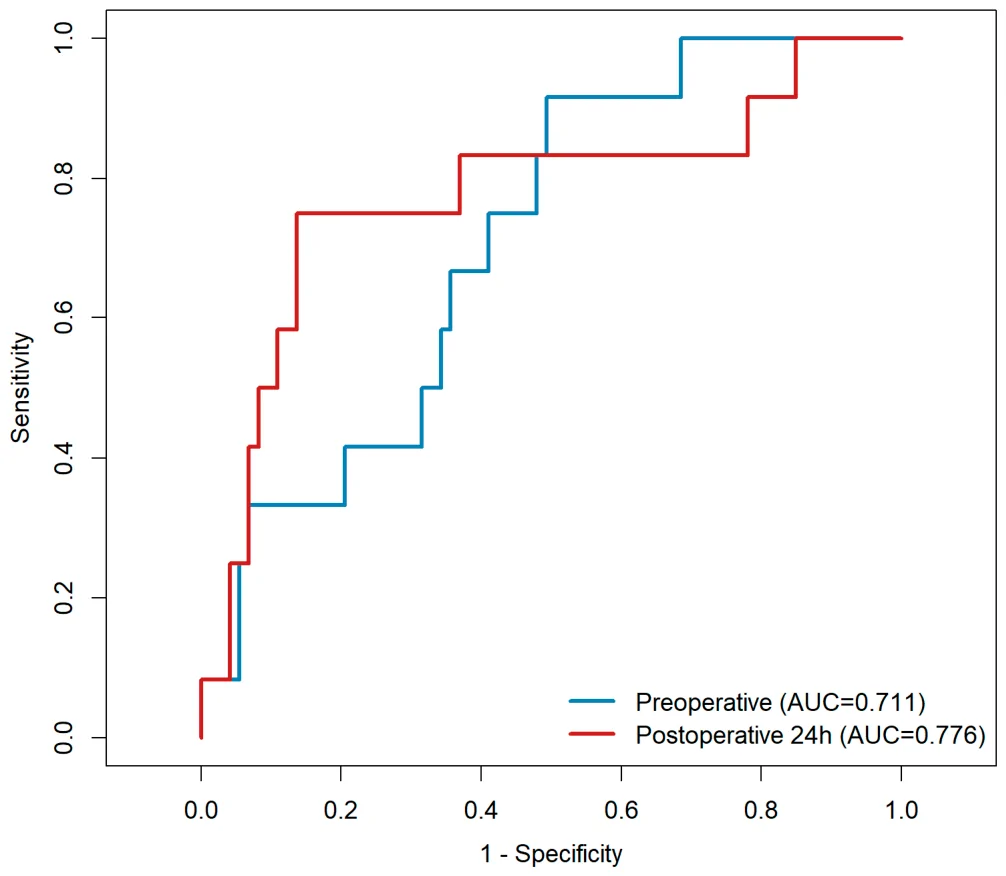
Receiver operating characteristic curves for the preoperative and postoperative 24-h models. The T1 model included baseline hemoglobin and IL-10 and had an apparent AUC of 0.711 and a bootstrap optimism-corrected AUC of 0.695. The T2 model included IL-10 and magnesium measured at 24 h and had an apparent AUC of 0.776 and a bootstrap optimism-corrected AUC of 0.762. The apparent AUCs did not differ significantly by DeLong’s test (*p* = 0.526). AUC, area under the curve; IL-10, interleukin-10.

A preoperative model including baseline hemoglobin and baseline IL-10 (T1) was compared with IL-10 (T2) and magnesium (T2). The preoperative model showed an apparent AUC of 0.711 and a bootstrap optimism-corrected AUC of 0.695, whereas the postoperative model showed an apparent AUC of 0.776 and a bootstrap optimism-corrected AUC of 0.762 (Figure 6). Although the postoperative model demonstrated higher discrimination, the difference between the apparent AUCs was not statistically significant by DeLong’s test (*p* = 0.526). To facilitate clinical interpretation of the selected predictors, univariable odds ratios and optimal Youden-based cutoff values were also estimated (Table 5). Among the individual predictors, IL-10 at 24 h showed the strongest univariate association with POAF (OR = 1.83, 95% CI: 1.27–2.85, *p* = 0.003), with an optimal cutoff of 5.80 pg/mL. Magnesium measured at 24 h was not independently associated with POAF in univariable analysis (*p* = 0.408). Nevertheless, it remained part of the final multivariable model, suggesting that its contribution depended on its combined effect with IL-10 rather than on its isolated association.

As an additional exploratory analysis, predictive performance was evaluated separately across four predefined predictor domains.

**Figure 7:**
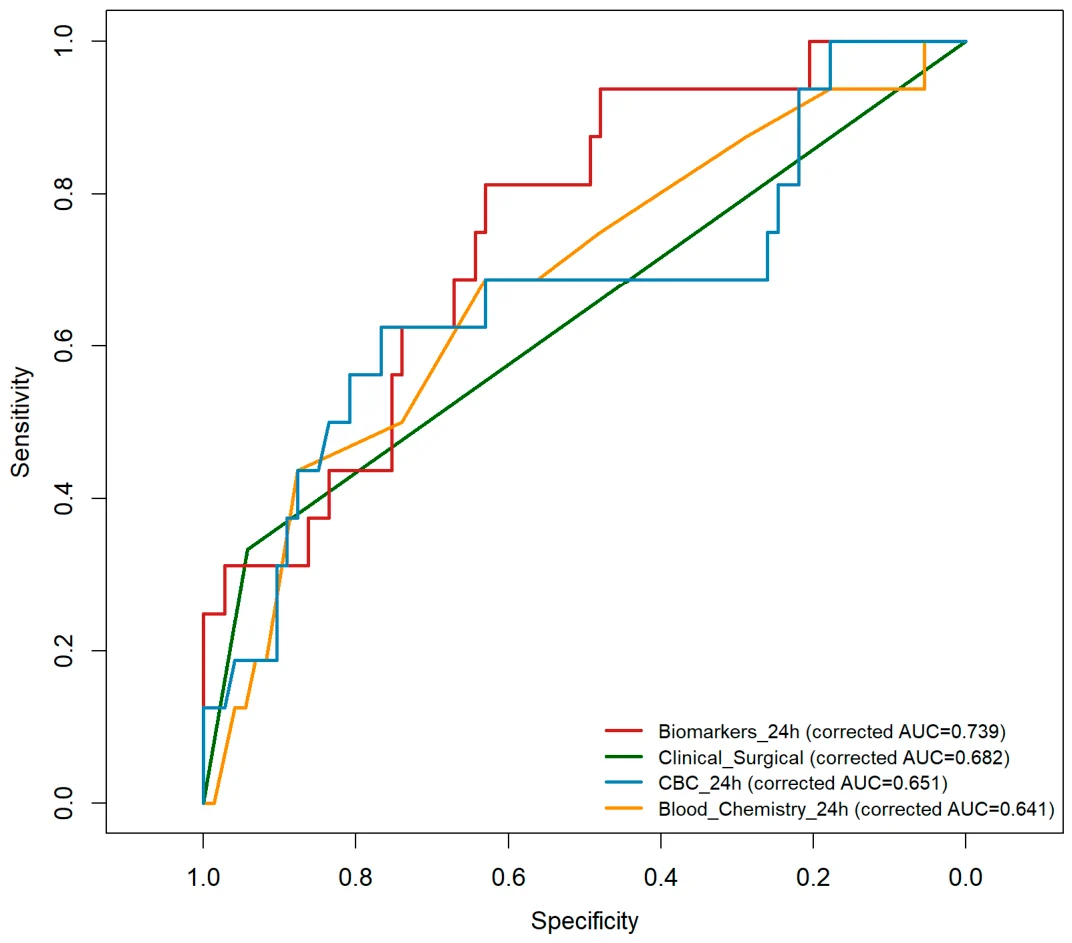
Receiver operating characteristic curves for predictor-domain models. Predictor selection was repeated within each domain using 1000 bootstrap replicates. The bootstrap optimism-corrected AUC was 0.739 for the inflammatory biomarker domain (IL-6, IL-8, and IL-10 at T2), 0.682 for the clinical-surgical domain (hypothyroidism), 0.652 for the complete blood count domain (hematocrit and platelet count at T2), and 0.639 for the blood chemistry domain (chloride at T2). AUC = area under the curve; IL = interleukin.

Among the four predictor domains, inflammatory biomarkers showed the highest discrimination, with a bootstrap optimism-corrected AUC of 0.739. The corresponding optimism-corrected AUCs were 0.682 for the clinical-surgical domain, 0.652 for the complete blood count domain, and 0.639 for the blood chemistry domain (Figure 7). Detailed performance metrics are presented in Supplementary Table S6.

### 3.7. Sensitivity Analyses

To assess the impact of surgical heterogeneity, a sensitivity analysis excluding patients undergoing combined CABG–valvular surgery was conducted (N = 73; 9 POAF events). In this restricted cohort, the association between log_2_-transformed IL-10 concentration at T2 and POAF remained statistically significant (OR = 1.71, 95% CI: 1.09–2.89; *p* = 0.020). The corresponding two-predictor model had an apparent AUC of 0.802 (95% CI: 0.629–0.975), though this estimate should be interpreted cautiously given the limited number of outcome events. Adjusting for surgical procedure type yielded similar results. The association between log_2_-transformed IL-10 concentration at T2 and POAF remained essentially unchanged (OR = 1.81; *p* = 0.0009). Relative to combined surgery, neither isolated CABG (*p* = 0.072) nor isolated valvular surgery (*p* = 0.704) was independently associated with POAF.

Additional sensitivity analyses were conducted to assess the potential influence of perioperative medication use. Beta-blockers, antiarrhythmic drugs, diuretics, corticosteroids, and statins were added individually to the final model in separate analyses (N = 85; 12 POAF events). Including these variables produced no material change in the estimated effects of IL-10 (OR range: 1.76–1.87) or magnesium (OR range: 3.10–3.54) compared with the primary model (Table 3). None of the medication variables was significantly associated with POAF (all *p* > 0.15; Supplementary Table S7). Parameter estimates for the three-predictor sensitivity model are presented in Supplementary Table S8. Overall, the sensitivity analyses demonstrated that the estimated association between IL-10 (T2) concentration and POAF remained robust across multiple alternative model specifications. Beyond the prespecified sensitivity analyses, several additional predictors were explored for their potential biological or clinical relevance.

### 3.8. Additional Exploratory Predictors

Several predictors demonstrated potential associations with POAF but were not retained in the final prediction model because they did not meet the predefined stability or sample-size criteria. Hypothyroidism showed the strongest exploratory clinical association with POAF in univariable analysis (OR = 8.13; *p* = 0.005) and was selected in 80.2% of bootstrap stability-selection resamples. However, because only nine patients had hypothyroidism, the corresponding regression estimates were considered insufficiently stable for inclusion in the primary prediction model. IL-6 concentration at 48 h also showed evidence of a possible association with POAF in univariable analysis (OR = 1.28; *p* = 0.051). However, when incorporated into the exploratory three-predictor sensitivity model (Supplementary Table S8), IL-6 remained non-significant (*p* = 0.109), providing no evidence that its inclusion improved the primary model. Accordingly, neither hypothyroidism nor IL-6 measured at 48 h was retained in the final primary prediction model.

## 4. Discussion

The overall incidence of postoperative atrial fibrillation (POAF) in our cohort was 17.9%, at the lower end of the 20–40% range reported after cardiac surgery with cardiopulmonary bypass (CPB) [6]. This lower incidence aligns with the cohort composition, which was predominantly composed of patients undergoing coronary artery bypass grafting and isolated valve surgery. Furthermore, stratification by procedure type (CABG 8.3%, valve surgery 22.5%, and combined surgery 30.8%) reproduced the risk gradient previously described by Mariscalco et al. (2014) [31], supporting the consistency of our findings with the available evidence.

Among the clinical variables evaluated, hypothyroidism showed a significant association with POAF (31.3% vs. 5.5%; *p* = 0.0018) and had the highest selection frequency for stability in the bootstrap analysis (80.2%). This finding is consistent with previous studies documenting a higher frequency of atrial arrhythmias in patients with thyroid disorders [32,33,34]. Despite its high bootstrap selection frequency (80.2%), hypothyroidism was not retained in the primary model because only nine patients had this condition, precluding reliable estimation of its independent contribution.

Patients who developed POAF had lower hemoglobin (T2) concentrations (9.80 vs. 10.8 g/dL; *p* = 0.007), a finding consistent with reports by Miceli et al. (2014) [35] and Türkkolu et al. (2021) [36]. However, given the sample size and the exploratory nature of the study, it is not possible to determine whether this association reflects an independent effect or simply greater perioperative physiological stress rather than a direct mechanistic association with POAF.

IL-6 measured at 24 h was the only biomarker that remained statistically significant after FDR correction, consistent with previous reports identifying IL-6 as one of the inflammatory mediators most consistently linked to POAF [20,36,37].

However, because predictor selection relied on bootstrap-based stability rather than statistical significance alone, IL-6 was not included in the final multivariable model.

The most relevant finding from the multivariable analysis was that IL-10 remained in the final model even after adjusting for surgical procedure type (OR = 1.81; *p* = 0.0009). Previous studies have shown that the IL-10 response during the first 24–48 h after CPB is associated with the magnitude of the systemic inflammatory response [20,37]. IL-10 is generally regarded as a compensatory anti-inflammatory cytokine. Accordingly, IL-10 may serve as a surrogate marker of the overall postoperative inflammatory response rather than as a direct causal mediator of POAF.

Magnesium was also retained in the final model (OR = 3.15; 95% CI: 0.90–11.88), providing complementary predictive information even though it did not reach significance in the univariable analysis. This result is consistent with evidence that magnesium modulates atrial electrical stability [38,39], but it should not be interpreted as evidence of therapeutic benefit, especially given the inconsistent results of prophylactic supplementation trials [40].

A particularly relevant contribution of this study was that a model based exclusively on preoperative variables (baseline hemoglobin and baseline IL-10) achieved a bootstrap optimism-corrected AUC of 0.681, whereas the postoperative model showed a corrected AUC of 0.728. The difference between the two models was not significant by DeLong’s test (*p* = 0.474). These findings suggest that a substantial proportion of POAF susceptibility may already be identifiable before surgery, despite most currently available risk scores relying predominantly on clinical variables and cardiovascular history [41,42].

A major strength of this study is the rigorous methodological framework used for model development and internal validation. The model’s construction and internal validation followed the TRIPOD guidelines [43] and incorporated predictor selection and optimism correction via bootstrap resampling, a strategy particularly suitable for small exploratory cohorts [44,45]. The final model demonstrated moderate discrimination (corrected AUC 0.728), acceptable calibration (Hosmer–Lemeshow *p* = 0.801), and low overall error (Brier score = 0.103). The corrected AUC observed in this exploratory cohort suggests acceptable discriminatory performance for an exploratory prediction model. Nevertheless, external validation in independent cohorts is required before its performance can be directly compared with established prediction models, including the nomogram developed by Zhu et al. (2023) [46] and the prospective validation reported by Cameron et al. (2017) [41].

The high negative predictive value (95.5% at the Youden cutoff point) is noteworthy because it indicates a good ability to identify patients with a low probability of POAF, whereas the moderate positive predictive value (47.4%) reflects the persistent difficulty in accurately predicting which patients will develop the event.

Among the study limitations, the single-center design, the moderate sample size (*n* = 89), and the limited number of events (16 POAF cases) must be acknowledged; these constraints restricted the number of variables that could be robustly evaluated and justified the construction of a parsimonious model with two predictors. Consequently, only a limited number of predictors could be reliably incorporated into the final model. These characteristics inherently limited model complexity. Although internal validation via bootstrap allowed for the correction of optimism and the exploration of the stability of the selected predictors, the developed models should be interpreted as exploratory and hypothesis-generating tools. Therefore, they will require external validation in independent prospective cohorts before being considered for clinical implementation.

Taken together, our results show that integrating clinical variables and inflammatory biomarkers enabled the development of a model with moderate predictive performance, adequate calibration, and optimism correction via bootstrapping. These findings suggest the potential value of perioperative inflammatory assessment for risk stratification of POAF after cardiac surgery with CPB and establish a solid methodological foundation for future multicenter validations and direct comparisons with established risk scores.

In addition, decision curve analysis suggested potential clinical utility across clinically relevant probability thresholds, though these findings require external validation.

## 5. Conclusions

The integration of perioperative inflammatory biomarkers with routine clinical variables enabled the development of a parsimonious predictive model with acceptable internal validity for postoperative atrial fibrillation after cardiac surgery with cardiopulmonary bypass. The model showed moderate discrimination after internal bootstrap validation and a high negative predictive value. The inclusion of IL-10 and magnesium in the final model suggests that they provide additional predictive value for postoperative atrial fibrillation beyond routine clinical variables. Although the results must be interpreted within the context of an exploratory cohort without external validation, they provide a methodological basis for future prospective, multicenter validation studies and for comparison with established clinical prediction models.

## Figures and Tables

**Table 1:** Baseline demographic and clinical characteristics according to postoperative atrial fibrillation status.

Variable	POAF Group (*n* = 16)	Non POAF Group (*n* = 73)	*p*-Value
Anthropometric data			
Age (years) ^b^	67 (50–76)	65 (50–78)	0.684
Male ^a^	10 (62.5%)	51 (69.9)	0.574
Female ^a^	6 (37.5)	22 (30.1)	
BMI (kg/m^2^) ^b^	26.8 (19.7–31.9)	27.1 (19.71–39.0)	0.589
Comorbidities			
Previous myocardial infarction ^a^	4 (25)	20 (27.4)	0.872
Type 2 diabetes mellitus ^a^	6 (37.5)	37 (50.7)	0.339
Systemic arterial hypertension ^a^	13 (81.3)	52 (71.3)	0.474
Obesity ^a^	4 (25)	20 (27.4)	0.847
Dyslipidemia ^a^	3 (18.75)	24 (32.9)	0.266
Hypothyroidism ^a^	5 (31.3)	4(5.5)	**0.0018**

Continuous variables are presented as median (range), and categorical variables as number and percentage, *n* (%). Between-group comparisons were performed using the Mann–Whitney U test for continuous variables and the chi-square test or Fisher’s exact test for categorical variables, as appropriate. Statistically significant *p*-values are shown in bold. BMI, body mass index; POAF, postoperative atrial fibrillation. BMI = body mass index; POAF = postoperative atrial fibrillation. ^a^ Chi-square test or Fisher’s; frequencies and percentages. ^b^ Mann–Whitney U test; median (range). *p* < 0.05 was considered statistically significant.

**Table 2:** Surgical, hematologic, and biochemical characteristics according to postoperative atrial fibrillation status.

Variable		POAF Group (*n* = 16)	Non POAF Group (*n* = 73)	*p*-Value
Surgical data				
Cardiopulmonary bypass time (min) ^b^		123 (73–217)	120 (64–255)	0.712
Aortic cross-clamp time (min) ^b^		91 (60–193)	92 (24–202)	0.634
Initial ACT (s) ^b^		115 (79–143)	118 (76–171)	0.352
Final ACT (s) ^b^		115 (103–138)	116 (84–167)	0.881
Number of grafts ^a^	0	9 (56.3)	31 (42.5)	-
	1	3 (18.8)	6 (8.2)	
	2	-	8 (11.0)	
	3	4 (25)	15 (20.5)	
	4	-	12 (16.4)	
	5	-	1 (1.4)	
Number of repaired valves ^a^	0	4 (25%)	33 (45.2%)	-
	1	10 (62.5%)	32 (43.8%)	
	2	1 (6.3%)	6 (8.25%)	
	3	1 (6.3%)	1 (2.7%)	
Pre-CPB blood loss (mL) ^b^		160 (15–300)	200 (20–400)	0.286
Intra-CPB blood loss (mL) ^b^		132.5 (0–400)	100 (0–640)	0.430
Post-CPB blood loss (mL) ^b^		325 (165–615)	300 (0–800)	0.348
EuroSCORE II (%) ^b^		1.36 (0.50–8.84)	1.09 (0.50–11.37)	0.168
Left ventricular ejection fraction (LVEF, %) ^b^		53 (20–73)	55 (24–86)	0.608
Blood count and biochemical parameters				
Hemoglobin (g/dL), T1 ^b^		13.75 (10.20–16.6)	14.20 (8.80–18.80)	0.175
Hemoglobin (g/dL), T2 ^b^		9.80 (6.20–12.10)	10.8 (5.60–15)	**0.007**
Hematocrit (%), T1 ^b^		39.35 (31–50)	41 (26.8–58.1)	0.148
Hematocrit (%), T2 ^b^		30 (21.1–42)	32.75 (24.3–51.4)	0.061
Platelets (×10^3^/µL), T1 ^b^		202 (136–352)	210 (37–471)	0.560
Platelets (×10^3^/µL), T2 ^b^		182 (124–301)	181 (75–345)	0.638
Leukocytes (×10^3^/µL), T1 ^b^		6.5 (3.50–9.25)	6.8 (2.5–17.20)	0.361
Leukocytes (×10^3^/µL), T2 ^b^		11.6 (7.87–23.70)	12.30 (1.60–23)	0.795
Glucose (mg/dL), T1 ^b^		96 (75–156)	101 (75–171)	0.426
Glucose (mg/dL), T2 ^b^		176 (85–589)	148 (75–324)	0.478
Urea (mg/dL), T1 ^b^		29 (20–65)	31 (17–66)	0.915
Urea (mg/dL), T2 ^b^		38 (21.6–95.9)	34 (16.8–93)	0.566
Creatinine (mg/dL), T1 ^b^		0.85 (0.50–1.36)	0.80 (0.51–1.40)	0.940
Creatinine (mg/dL), T2 ^b^		0.85 (0.60–1.33)	0.9 (0.51–2.30)	0.696

Continuous variables are presented as median (range), and categorical variables as counts and percentages (*n*, %). Comparisons were performed using the Mann–Whitney U test for continuous variables and the chi-square test or Fisher’s exact test for categorical variables, as appropriate. Statistically significant *p*-values are shown in bold. ACT = activated clotting time; CPB = cardiopulmonary bypass; LVEF = left ventricular ejection fraction; POAF = postoperative atrial fibrillation; preoperative, measurement obtained before surgery; T1 = Patient plasma sampling at preoperatory; T2 = Patient plasma sampling at 24 h postoperative. ^a^ Chi-square test or Fisher’s; frequencies and percentages. ^b^ Mann–Whitney U test; median (range). In bold, the only variable that was considered statistically significant.

**Table 3:** Firth bias-reduced multivariable logistic regression model for postoperative atrial fibrillation.

Variable	β (SE)	OR	95% CI	*p*-Value
Intercept	−5.630 (1.652)	0.0036	0.00009–0.0836	0.0005
log2(IL-10, 24 h)	0.585 (0.214)	1.79	1.20–2.90	0.0046
Magnesium (24 h, mEq/L)	1.149 (0.621)	3.15	0.90–11.88	0.0717

The model included 89 patients and 16 POAF events. IL-10 concentrations were transformed as log_2_(IL-10 + 1); therefore, the corresponding odds ratio represents the change in POAF odds associated with an approximate doubling of IL-10 concentration.

**Table 4:** Diagnostic classification performance of the final model at the Youden-optimal predicted-probability threshold.

Metric	Estimate
Youden threshold	0.191
Sensitivity	75.0%
Specificity	86.3%
PPV	47.4%
NPV	95.5%

The threshold was selected to maximize the Youden index in the development cohort. NPV = negative predictive value; PPV = positive predictive value.

**Table 5:** Univariate odds ratios and optimal Youden-based cutoff values for individual predictors of postoperative atrial fibrillation.

Variable	N	OR (95% CI)	*p*-Value	AUC	Optimal Cutoff
IL-10 (T2)	89	1.83 (1.27–2.85)	0.003	0.739	>5.80 pg/mL
Magnesium (T2)	89	1.64 (0.47–5.23)	0.408	0.522	>2.28 mEq/L
Hemoglobin (T1)	89	0.75 (0.54–1.02)	0.068	0.673	<14.15 g/dL
IL-10 (T1)	89	1.54 (0.90–2.62)	0.107	0.672	>0.58 pg/mL

Continuous predictors were dichotomized at the threshold that maximized the Youden index (sensitivity + specificity − 1) in univariate receiver operating characteristic analysis. IL-10 concentrations were log2-transformed for modeling and are presented here in their original units (pg/mL) for clinical interpretability. OR, odds ratio; CI, confidence interval; AUC, area under the curve.

## Data Availability

The original contributions presented in this study are included in the article/Supplementary Material. Further inquiries can be directed to the corresponding author.

## Supplementary Materials

The following supporting information can be downloaded at: https://www.mdpi.com/article/10.3390/medsci14050513/s1, Table S1: Incidence of postoperative atrial fibrillation according to surgical procedure; Table S2: Comparative analysis of inflammatory biomarkers in different pre and postoperative times and surgery types; Table S3: Perioperative inflammatory biomarker concentrations according to postoperative atrial fibrillation status; Table S4: Univariable logistic regression screening of candidate predictors for postoperative atrial fibrillation; Table S5: Stability selection of candidate predictors across bootstrap-resampled LASSO models; Table S6: Internal validation of predictor-domain models; Table S7: Sensitivity analysis: main model adjusted individually for perioperative pharmacologic treatments; Table S8: Three-predictor sensitivity model.

## Abbreviations

ACT	Activated Clotting Time
AMI	Acute Myocardial Infarction
AUC	Area Under the Curve
BMI	Body Mass Index
CABG	Coronary Artery Bypass Grafting
CCI	Chronic Cardiac Insufficiency
CCS	Chronic Coronary Syndrome
CPB	Cardiopulmonary Bypass
CRP	C-Reactive Protein
CVDs	Cardiovascular Diseases
Euro SCORE II	European System for Cardiac Operative Risk Evaluation II
IL-6	Interleukin-6
IL-8	Interleukin-8
IL-10	Interleukin-10
IQR	Interquartile Range
LVEF	Left Ventricular Ejection Fraction
POAF	Postoperative Atrial Fibrillation
ROC	Receiver Operating Characteristic
SAH	Systemic Arterial Hypertension
STS score	Society of Thoracic Surgeons score
T2DM	Type 2 Diabetes Mellitus
Valve	Valve replacement surgery
