# Supplementary material for: Exploratory Modeling of Postoperative Atrial Fibrillation After Cardiac Surgery with Cardiopulmonary Bypass Using Inflammatory Biomarkers and Clinical-Surgical Factors
Source: Med Sci (Basel). 2026 Aug 25;14(5):513. doi: 10.3390/medsci14050513 (PMC13609514; doi:10.3390/medsci14050513)
Supplement: Supplementary file 1 [file medsci-14-00513-s001.zip › medsci-4449070-supplementary.pdf]

Table S1. Incidence of postoperative atrial fibrillation according to surgical procedure

Surgical procedure	Total patients	POAF	Incidence (%)
CABG	36	3	8.3
Valve surgery	40	9	22.5
Combined CABG + valve	13	4	30.8

POAF=postoperative atrial fibrillation; CABG=coronary artery bypass grafting. The incidence was calculated as the number of patients who developed POAF divided by the total number of patients in each surgical procedure group, expressed as a percentage.

Table S2. Comparative analysis of inflammatory biomarkers in different pre and postoperative times and surgery types.

**Variable**	**CABG group (*n*= 32)**	**Valve group (*n* = 39)**	***p-Value***
Proinflammatory interleukins			
Preoperative IL-8 at T1 (pg/mL)	4.26 (0.58-13.2)	6.52 (2.66-143.34)	**0.01**
Postoperative IL-8 at T2 (pg/mL)	11.29 (2.42-83.9)	12.78 (1.88-340.65)	0.216
Postoperative IL-8 at T3 (pg/mL)	8.35 (0.36-451.17)	10.33 (2.27-119.16)	0.272
Preoperative IL-10 at T1 (pg/mL)	0.41 (0-6.62)	0.73 (0-8.69)	**0.027**
Postoperative IL-10 at T2 (pg/mL)	2.68 (0.19-194.0)	2.27 (0-133.63)	0.909
Postoperative IL-10 at T3 (pg/mL)	1.57 (0-328.78)	1.10 (0-19.6)	0.831
Preoperative IL-6 at T1 (pg/mL)	0.19 (0-11.34)	0.43 (0-10.63)	0.068
Postoperative IL-6 at T2 (pg/mL)	4.83 (0-158.37)	3.90 (0-479.42)	0.835
Postoperative IL-6 at T3 (pg/mL)	2.69 (0-182.5)	2.03 (0.11-83.59)	0.279
Preoperative CRP at T1 (mg/L)	1.92 (0.71-13.21)	1.47 (0.68-50.04)	0.696
Postoperative CRP at T2 (mg/L)	113.4 (1.88-123.28)	98.36 (11.9-123.51)	**0.050**
Postoperative CRP at T3 (mg/L)	123 (1.85-128.74)	123.35 (65.16-127.87)	0.519

CABG = coronary artery bypass grafting; IL = interleukin; CRP = C-reactive protein; T1 = preoperative; T2 and T3 = postoperative time points at 24 h and 48 h respectively. Comparisons between the CABG and valve surgery groups were performed using the Mann–Whitney U test. Statistically significant adjusted *p*-values are shown in bold. Significance value < 0.05.

Table S3. Perioperative inflammatory biomarker concentrations according to postoperative atrial fibrillation status.

Variable	POAF Group (n = 16)	Non POAF Group (n= 73)	Participants analyzed, POAF / No POAF	p-Value	FDR p-Value
IL-8 at T1 (pg/mL)	6.73 (3.6-143.3)	5.35 (0.5-34.3)	16 / 73	0.205	0.312
IL-8 at T2 (pg/mL)	21.85 (5.3-512.1)	11.02 (1.8-127.3)	12 / 73	**0.010****	0.063
IL-8 at T3 (pg/mL)	10.09 (5.0-169.3)	9.2 (0.36-539.2)	9 / 73	0.171	0.103
IL-10 at T1 (pg/mL)	1.10 (0-8.19)	0.43 (0-8.95)	16 / 73	**0.029***	0.329
IL-10 at T2 (pg/mL)	7.33 (0.19-194.0)	2.13 (0-67.39)	12 / 73	**0.003****	0.063
IL-10 at T3 (pg/mL)	1.52 (0-21.97)	1.56 (0-328.7)	9 / 73	0.389	0.312
IL-6 at T1 (pg/mL)	0.98 (0-16.73)	0.35 (0-16.72)	16 / 73	0.174	0.100
IL-6 at T2 (pg/mL)	12.44 (1.44-1102)	4.97 (0-158.3)	12 / 73	**0.012***	**0.046***
IL-6 at T3 (pg/mL)	13.56 (0.11-131.5)	2.42 (0-202.5)	9 / 73	**0.038***	0.507
CRP at T1 (mg/L)	1.75 (0.75-50)	1.92 (0.68-31.94)	16 / 73	0.894	0.958
CRP at T2 (mg/L)	101.8 (5.65-123.51)	105.4 (1.88-125)	12 / 73	0.957	0.962
CRP at T3 (mg/L)	124 (37.15-127.6)	122.8 (1.85-128.7)	9 / 73	0.761	0.874

Values are presented as the median (range). POAF = postoperative atrial fibrillation; IL = interleukin; T1 = preoperative; T2 and T3 = postoperative time points at 24 h and 48 h, respectively; FDR = false discovery rate. Comparisons at T2 and T3 were limited to patients who remained free of POAF before blood collection at the corresponding landmark time. Between-group comparisons were performed using the Mann–Whitney U test. FDR-adjusted p-values were calculated using the Benjamini–Hochberg procedure. Statistically significant adjusted p-values are shown in bold. ** Significance value < 0.01; * Significance value < 0.05.

Table S4. Univariable logistic regression screening of candidate predictors for postoperative atrial fibrillation

**Predictor**	**N**	**OR**	**95% CI**	***p*-Value**	**AUC**
IL-10 (T2), log_2	89	1.83	1.27–2.85	**0.0027****	0.739
IL-8 (T2), log_2	89	1.70	1.19–2.55	**0.0058****	0.705
IL-6 (T2), log_2	89	1.45	1.12–1.94	**0.0062****	0.702
Hematocrit (T1)	89	0.88	0.77–0.96	**0.0212***	0.699
IL-6 (T3), log_2	89	1.28	1.00–1.64	0.0511	0.666
Chloride (T2)	89	1.19	1.01–1.43	0.0531	0.682
Hematocrit (T2)	89	0.88	0.76–1.00	0.0622	0.646
Hemoglobin (T1)	89	0.75	0.54–1.02	0.0684	0.673
Platelets (T2)	89	0.99	0.98–1.00	0.0855	0.656
IL-8 (T1), log_2	89	1.58	0.91–2.84	0.1036	0.602
IL-10 (T1), log_2	89	1.54	0.90–2.61	0.1072	0.672
EuroSCORE II	89	1.27	0.95–1.77	0.1082	0.610
Number of grafts	89	0.74	0.48–1.06	0.1239	0.608
Glucose	89	1.02	0.99–1.04	0.1404	0.616
Chloride	89	1.14	0.97–1.36	0.1406	0.622
BMI	89	0.90	0.76–1.04	0.1757	0.578
IL-8 (T3), log_2	89	1.24	0.89–1.71	0.1812	0.610
Leukocytes	89	1.17	0.92–1.50	0.1936	0.524
Pre-CPB bleeding	89	1.00	0.99–1.00	0.2238	0.585
Magnesium	89	3.04	0.42–22.20	0.2445	0.650
Post-CPB bleeding	89	1.00	1.00–1.01	0.3006	0.575
Magnesium (T2)	89	1.64	0.47–5.23	0.4078	0.522

IL-6 (T1), log_2	89	1.20	0.75–1.86	0.4109	0.609
Left ventricular ejection fraction	89	0.17	0.002–12.51	0.4212	0.541
Aortic cross-clamp time	89	1.01	0.99–1.02	0.4671	0.454
Calcium	89	0.75	0.31–1.75	0.5096	0.559
IL-10 (T3), log_2	89	1.11	0.78–1.52	0.5218	0.429
Intra-CPB bleeding	89	1.00	1.00–1.01	0.5373	0.563
Urea	89	1.01	0.97–1.06	0.5570	0.539
Hemoglobin (T2)	89	0.93	0.72–1.25	0.5875	0.561
Potassium (T2)	89	0.77	0.26–2.09	0.6110	0.535
Cardiopulmonary bypass time	89	1.00	0.99–1.01	0.6276	0.536
Platelets	89	1.00	0.99–1.01	0.6287	0.533
Urea (T2)	89	1.01	0.97–1.04	0.6824	0.555
Creatinine	89	0.63	0.05–7.01	0.7108	0.534
Leukocytes (T2)	89	0.98	0.86–1.10	0.7206	0.505
Creatinine (T2)	89	0.71	0.08–4.32	0.7275	0.509
Age	89	1.01	0.94–1.09	0.7914	0.528
Sodium	89	0.99	0.86–1.14	0.8437	0.499
CRP (T3), log_2	89	1.07	0.50–5.19	0.8821	0.524
Glucose (T2)	89	1.00	0.99–1.01	0.9101	0.516
Potassium	89	1.01	0.28–3.63	0.9838	0.514
CRP (T1), log_2	89	1.01	0.55–1.66	0.9840	0.489
CRP (T2), log_2	89	1.00	0.62–1.96	0.9982	0.496

Candidate predictors were individually evaluated using univariable logistic regression. Biomarker concentrations were log_2-transformed before analysis. Odds ratios for log_2-transformed biomarkers represent

the estimated change in the odds of postoperative atrial fibrillation per approximately doubling of biomarker concentration. AUC = area under the receiver operating characteristic curve; BMI = body mass index; CI = confidence interval; CPB = cardiopulmonary bypass; CRP = C-reactive protein; T1 = preoperative; T2 and T3 = postoperative time points at 24 h and 48 h, respectively. Statistically significant adjusted *p*-values are shown in bold. **Significance value < 0.01; * Significance value < 0.05.

Table S5. Stability selection of candidate predictors across bootstrap-resampled LASSO models

Predictor	Selection frequency (%)	Positive coefficient (%)	Final decision	Reason for final decision
Hypothyroidism	80.2	97.1	Excluded	Very small exposed subgroup (n = 9)
IL-10 (T2)	66.4	89.9	Included	Stable selection, temporally valid, biologically plausible
IL-6 (T3)	65.4	97.4	Sensitivity model	Later postoperative measurement
IL-8 (T2)	54.0	89.4	Excluded	Lower stability than retained predictors
Magnesium (T2)	49.7	75.7	Included	Stable postoperative metabolic marker

Selection frequencies were obtained from 300 bootstrap-resampled LASSO models. Final predictor retention was also assessed for temporal validity, subgroup size, biological plausibility, and coefficient stability. Selection frequency is the percentage of bootstrap models in which each predictor was retained, whereas a positive coefficient indicates the percentage of selected models in which the predictor showed a positive association with POAF. IL = interleukin; LASSO = least absolute shrinkage and selection operator; POAF = postoperative atrial fibrillation; T2 and T3 = postoperative time points at 24 h and 48 h, respectively.

Table S6. Internal validation of predictor-domain models

Predictor domain	Selected predictors	N	POAF events	Apparent AUC	Optimism	Corrected AUC
Clinical–surgical	Hypothyroidism	89	16	0.756	0.074	0.682
Inflammatory biomarkers	IL-6, IL-8, IL-10 (T2)	85	12	0.835	0.096	0.739
Blood chemistry	Chloride (T2)	85	12	0.724	0.085	0.639
Complete blood count	Hematocrit (T1) + platelets (T2)	85	12	0.744	0.092	0.652

Predictor selection was repeated within each predictor domain during bootstrap validation. Apparent AUC represents model performance on the original dataset, whereas optimism reflects the estimated overestimation of performance due to bootstrap resampling. Corrected AUC is the optimism-corrected estimate of model discrimination. AUC = area under the receiver operating characteristic curve; IL = interleukin; POAF = postoperative atrial fibrillation; T1 = preoperative; T2 = postoperative time point at 24 h.

Table S7. Sensitivity analysis: main model adjusted individually for perioperative pharmacologic treatments.

**Treatment**	**OR Treatment [IC]**	***p-Value***	**AUC set [IC]**
Beta blockers	0.80 (0.13–9.54)	0.830	0.774 (0.601–0.947)
Antiarrhythmic drugs	1.03 (0.24–6.02)	0.968	0.775 (0.601–0.950)
Diuretic	0.48 (0.11–2.31)	0.341	0.779 (0.619–0.938)
Corticoesteroid	2.61 (0.41–13.35)	0.282	0.789 (0.613–0.964)
Statin	0.35 (0.08–1.52)	0.157	0.784 (0.631–0.938)

Each perioperative treatment (beta-blocker, antiarrhythmic drug, diuretic, corticosteroid, and statin) was added individually to the primary model ($\log_2$ IL-10 at T2 + magnesium at T2). IL-10 and magnesium odds ratios remained stable across all models (OR ranges: 1.76–1.87 and 3.10–3.54, respectively), consistent with the primary model. None of the treatment variables were independently associated with POAF (all $p > 0.15$). N = 85; POAF events = 12 for all models. AUC = area under the receiver operating characteristic curve; CI = confidence interval; IL = interleukin; OR = odds ratio; POAF = postoperative atrial fibrillation; T2 = postoperative time point at 24 h.

Table S8. Three-predictor sensitivity model

Predictor	β	OR	95% CI	*p*-Value
Intercept	6.158	0.002	0.00004–0.076	0.0009
IL-10 (T2), $\log_2$	0.514	1.67	1.09–2.71	**0.0184**
Magnesium (T2)	0.992	2.70	0.66–9.79	0.1517
IL-6 (T3), $\log_2$	0.249	1.28	0.95–1.77	0.1086

Sensitivity analysis using Firth bias-reduced logistic regression including IL-10 (T2), magnesium (T2), and IL-6 (T3). Odds ratios for $\log_2$-transformed biomarkers represent the change in POAF odds associated with an approximate doubling of biomarker concentration. CI = confidence interval; IL = interleukin; OR = odds ratio; POAF = postoperative atrial fibrillation; T2 and T3 = postoperative time points. Statistically significant adjusted *p*-values are shown in bold. Significance value < 0.05.
